# Cambios estructurales generados en el complejo craneofacial posterior a la expansión maxilar asistida con microimplantes del tipo maxilar skeletal expander (MSE). una revisión

**DOI:** 10.21142/2523-2754-1302-2025-243

**Published:** 2025-05-16

**Authors:** Diana Cecilia Zárate-Guerra, Gissella Gutiérrez-Tapia

**Affiliations:** 1 Division of Orthodontic, School of Dentistry, Universidad Cientifica del Sur. Lima, Peru. 100038556@cientifica.edu.pe, ggutierrezt@cientifica.edu.pe Universidad Científica del Sur Division of Orthodontic School of Dentistry Universidad Cientifica del Sur Lima Peru 100038556@cientifica.edu.pe ggutierrezt@cientifica.edu.pe

**Keywords:** expansión maxilar asistida con microimplantes, expansor esquelético maxilar, sutura medio palatina, Maxillary expansion assisted by micro-implants, maxillary skeletal expander, midpalatal suture

## Abstract

**Objetivo:**

La revisión tuvo como objetivo evaluar los cambios estructurales generados en el complejo craneofacial posterior a la expansión maxilar asistida con microimplantes MARPE (*miniscrew assisted rapid palatal expander*) en pacientes con deficiencia transversal maxilar.

**Materiales y métodos:**

En esta revisión se incluyeron dentro de la búsqueda, estudios que utilizaron el diseño de expansor maxilar esquelético MSE (*maxillary skeletal expander*), cuyo tornillo se encuentra en la parte posterior alineado con la apófisis cigomática, con apoyo en bandas de primeros molares superiores (U6) y 4 microimplantes paramediales a la sutura media palatina (SMP) que buscan un anclaje bicortical (tanto palatino como nasal). Se revisaron cuatro bases de datos para el estudio hasta julio de 2024 (PubMed, Scopus, Embase y ScienceDirect). Se seleccionaron estudios que reportaron cambios a nivel esquelético y dentoalveolar en pacientes con deficiencia transversal maxilar evaluados en tomografía computarizada de haz cónico (TCHC). La metodología fue garantizada con el uso de PRISMA *checklist*.

**Resultados:**

Después de realizar la búsqueda, se seleccionaron 13 artículos de acuerdo con los criterios de selección. Con respecto a los cambios esqueléticos, la disyunción en el corte axial es de forma paralela o casi paralela, tanto en medidas lineales como angulares (la espina nasal posterior representa desde el 82% hasta el 101% con respecto a espina nasal anterior). Dos estudios reportan en la mitad de la muestra una desviación en la disyunción a nivel de ENA de 1,1 mm (+/- 1mm) entre un lado y otro. El paralelismo depende de la desarticulación de la sutura pterigopalatina. Se reportó una correlación entre el anclaje bicortical y la apertura de dicha sutura (p = 0,0003) hasta en el 55,6% de pacientes evaluados. En el corte coronal, la expansión es piramidal con vértice superior y el fulcrum estaría ubicado en el punto más externo e inferior de la sutura frontocigomática, lo que permite una rotación de 0,6° del complejo cigomáticomaxilar por cada milímetro de expansión. En relación con los cambios dentoalveolares, los estudios encuentran en promedio una inclinación bucal de las primeras molares superiores de 3°.

**Conclusiones:**

La expansión maxilar asistida con microimplantes tiene un impacto significativo a nivel de las estructuras del complejo craneofacial. Además, al asegurar movimientos ortopédicos en pacientes jóvenes y jóvenes adultos, se reducen los efectos adversos a nivel dentario y periodontal que podrían generarse tras una expansión compensatoria o no esquelética. Se requieren estudios aplicados a poblaciones más amplias que aseguren los efectos mencionados.

## Abstract (en)

**Objective:**

The review aimed to assess the structural changes in the craniofacial complex following maxillary expansion assisted by micro-implants, known as the “*Miniscrew assisted rapid palatal expander*” (*MARPE*) in patients with maxillary transverse deficiency.

**Materials and Methods:**

The research included a specific type of expander called “Maxillary Skeletal Expander” (MSE) the design features a posteriorly positioned screw aligned with the zygomatic buttress, supported by four micro-implants located paramedial to the midpalatal suture with bicortical engagement of the palatal vault and the nasal floor. Four databases (PubMed, Scopus, Embase and ScienceDirect) were reviewed until July 2024. Studies reporting skeletal and dentoalveolar changes in patients with transverse maxillary deficiency evaluated using cone-beam computed tomography (CBCT) were selected. The methodology was ensured using the PRISMA checklist.

**Results:**

13 articles were selected according to the inclusion criteria. Regarding skeletal changes, the disjunction in the axial cut is parallel or nearly parallel, both in lineal and angular measurements (the posterior nasal spine represents between 82% and 101% in relation to anterior nasal spine) Two studies report a deviation in the disjunction at the ANS level of 1.1mm (+/- 1mm) in half of the sample, between one side and the other. Parallelism depends on the disarticulation of the pterygopalatine suture. A correlation was reported between bicortical anchorage anc the opening of this suture (P=0.0003) in 55.6% of the evaluated patients. In the coronal cut the expansion is pyramidal with an upper vertex, and the fulcrum would be located at the most external and inferior point of the frontozygomatic suture, allowing a rotation of 0.6° of the zygomaticomaxillary complex for each millimeter of expansion. Regarding dentoalveolar changes, studies find an average buccal inclination of the upper first molars of 3°.

**Conclusions:**

Maxillary expansion assisted by micro-implants significantly impacts craniofacial structures. Furthermore, by ensuring orthopedic movements in young and young adult patients, adverse dental and periodontal effects associated with compensatory or non-skeletal expansion are reduced. Further studies on larger populations are required to confirm these effects.

## INTRODUCCIÓN

La deficiencia transversal maxilar es una alteración que se origina durante el crecimiento e incrementa la severidad de las maloclusiones. La principal manifestación clínica de la deficiencia transversal maxilar es la presencia de mordida cruzada posterior (MCP). Su etiología es multifactorial, ya que puede deberse a alteraciones genéticas o factores ambientales que influyen durante el crecimiento de los maxilares, como los hábitos ^1^^,^^2^. La prevalencia de la deficiencia transversal maxilar tiene un rango del 4% al 17% ^3^^,^^4^ en pacientes con dentición decidua o mixta, y del 10% hacia la adultez ^5^^,^^6^.

La definición de “expansión maxilar” fue descrita en 1860 por Angell ^7^^,^^8^, quien propuso la posibilidad de ampliar el arco superior con aparatología fija. Este es adoptado hasta el día de hoy como el tratamiento ideal en edades tempranas. No obstante, se vuelve menos predecible en pacientes sin crecimiento residual, lo que puede generar inclinaciones bucales en los dientes de apoyo, dehiscencias o alteraciones periodontales. En adultos, el abordaje puede ser quirúrgico y se denomina SARPE (*surgically assisted rapid palatal expansion*) ^9^. Aun así, los pacientes suelen ser reacios en cuanto a realizarse cirugías, por ser una opción invasiva y costosa ^10^.

En 2010, Lee *et al*. ^11^ introdujo el concepto del uso de expansores maxilares asistidos por microimplantes MARPE (*miniscrew assisted rapid palatal expander*), una alternativa a la cirugía en pacientes jóvenes y adultos con deficiencia transversal maxilar. Desde entonces, diversos investigadores reportaron variaciones en diseño y protocolo ^12^^,^^13^. Sin embargo, no es posible generalizar los efectos tras la expansión con MARPE, pues las modificaciones develan efectos estructurales diferentes.

Woon Moon *et al*. ^14^, en 2014, desarrollaron el expansor esquelético maxilar MSE (*maxillary skeletal expander*, Biomaterial Korea Seul, South Korea), un diseño de MARPE que consistía en ubicar el tornillo lo más atrás posible, a la altura de la apófisis cigomática ^15^^,^^16^. Asimismo, constaba de brazos soldados a las bandas de los primeros molares superiores (U6) y cuatro microimplantes TAD (*temporal anchorage device*), colocados de forma paramedial al rafe medio del paladar, cuya bicorticalidad (palatina y nasal) asegura la trasmisión de fuerzas a las suturas de mayor resistencia: sutura media palatina (SMP), sutura cigomáticomaxilar y pterigopalatina ^17^^,^^18^. Posteriormente, se han realizado múltiples investigaciones en torno a este diseño, las cuales respaldan su uso en pacientes con y sin crecimiento residual, hiperdivergencia y obstrucción respiratoria ^15^^,^^18^^,^^19^^,^^28^^,^^29^.

Este estudio busca conocer y describir los cambios estructurales generados en el complejo craneofacial posterior a la expansión con MARPE, en específico con el MSE, a fin de obtener una mayor predictibilidad en el tratamiento de pacientes con deficiencia transversal maxilar. 

## MATERIALES Y MÉTODOS

**Figura 1:**
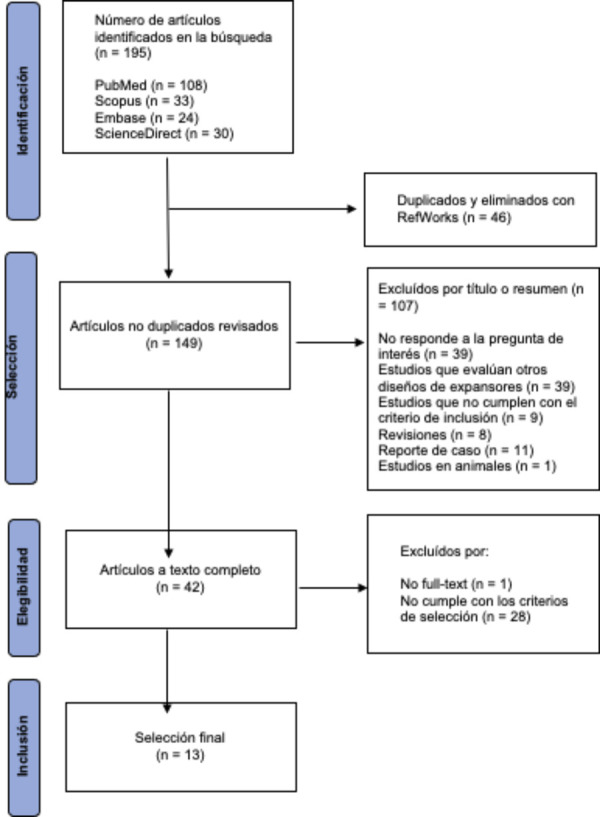
Diagrama de flujo PRISMA de la revisión de literatura

Esta revisión utilizó el diagrama PRISMA (Preferred Reporting Items for Systematic Reviews and Meta-Analyses) ^24^ para su metodología y para la selección de artículos (Figura 1).

### Estrategia de búsqueda

Se incluyeron artículos publicados hasta el 15 de julio de 2024 teniendo cuatro bases de datos principales: PubMed, Scopus, Embase y ScienceDirect. Se consideraron descriptores MeSH para la búsqueda (Medical Subject Heading) y palabras clave en los títulos donde se incluyeron operadores booleanos: (skeletal AND (effect OR assessment OR change)) AND ((microimplant assisted rapid palatal expansion) OR (maxillary skeletal expander)) AND (CBCT) y, finalmente, se gestionaron las referencias con RefWorks (Tabla 1).

**Tabla 1:** Estrategia de búsqueda de descriptores de las diferentes bases de datos

PubMed (15/03/2024)
n = 108
(skeletal AND (effect OR assessment OR change)) AND ((microimplant assisted rapid palatal expansion) OR (maxillary skeletal expander)) AND ((CBCT) OR (CONE BEAM COMPUTED TOMOGRAPHY) OR (3D IMAGINING))
Scopus (15/03/2024)
n = 33
TITLE-ABS-KEY ((skeletal AND (effect OR assessment)) AND ((microimplant AND assisted AND rapid AND palatal AND expansion) OR (maxillary AND skeletal AND expander)) AND (cbct)) AND (LIMIT-TO (DOCTYPE , "ar") ) AND (LIMIT-TO (SUBJAREA , "DENT"))
Embase (15/03/2024)
n = 24
AND ('bone borne rapid maxillary expander' OR 'bone supported device' OR 'cone beam computed tomography scanner'/dv OR 'expansion screw'/dv OR 'imaging software'/dv OR 'imaging system'/dv OR 'maxillary expander' OR 'maxillary skeletal expander' OR 'orthodontic device'/dv OR 'palatal expander'/dv OR 'suture'/dv)
ScienceDirect (15/03/2024)
n = 30
(skeletal AND (effect OR assessment)) AND ((microimplant assisted rapid palatal expansion) AND (maxillary skeletal expander)) AND (CBCT)

### Criterios de selección: estrategia P (población), I (intervención), C (comparación), O (*outcome* o resultado)

La búsqueda de literatura se estructuró utilizando la estrategia PICO: P (pacientes con deficiencia transversal maxilar sin crecimiento residual), I (expansor maxilar asistido por microimplantes tipo MSE), C (pre y postratamiento), O (efectos esqueléticos, periodontales y dentarios). Esto nos ayudó a definir procedimientos y acciones para obtener la información necesaria y responder de forma concreta la pregunta. Se incluyeron ensayos clínicos aleatorizados, cuasiexperimentales, estudios de cohorte y estudios de caso control. Se seleccionaron investigaciones en pacientes con deficiencia transversal maxilar que requerían corrección con expansor maxilar asistida con microimplantes, y se evaluaron los efectos del MARPE a nivel esquelético, periodontal y dentario. Los artículos debían estar publicados en español o inglés. Además, se inclueron estudios que valoraran los cambios estructurales en tomografía computarizada de haz cónico.

Los criterios de exclusión incluyeron reportes de caso, cartas al editor, editoriales, revisiones de literatura, revisiones sistemáticas o metaanálisis. Asimismo, se excluyeron estudios que evalúen pacientes con tratamiento previo de ortodoncia o cirugía maxilofacial, estudios en pacientes con malformaciones congénitas o craneofaciales, así como estudios en animales. 

### Riesgo de sesgo 

Se utilizó la herramienta ROBINS-I (*Risk Of Bias In Non-Randomized Studies - of Interventions*) ^25^ para estimar el riesgo de sesgo y la calidad de los artículos seleccionados. 

### Recolección de datos

Se seleccionaron 13 artículos y se obtuvo un 100% de concordancia en la extracción de resultados de interés. Las investigaciones fueron analizadas por la investigadora principal, quien recolectó la data de los estudios de mayor relevancia, evaluó inicialmente títulos y resúmenes, y seleccionó de acuerdo con los criterios de elegibilidad. En caso de dudas o discrepancias, estas fueron resueltas por la segunda autora. La lista de artículos y los motivos de exclusión fueron registrados de igual manera. 

### Resultados de interés

Los datos recopilados de los estudios fueron los siguientes: autores, país y año de publicación, diseño de estudio, tamaño de muestra, sexo, rango de edad y edad promedio, características clínicas, diseño de expansor y características de microimplantes, protocolo de activación, periodo y método de evaluación, resultados primarios y otros resultados reportados. Los datos fueron sintetizados según los resultados de interés (Tabla 2).

**Tabla 2:** Tabla de extracción de información

Id	Autor, país, año	Diseño de estudio	Tamaño de muestra (N, mujeres y hombre)	Rango de edad y edad promedio (años)	Carácterísticas clínicas	Diseño del expansor y características de los microimplantes	Protocolo de activación	Periodo y método de evaluación	Resultados
1	Cantarella *et al*. (^30^), EE. UU., 2017	Retrospectivo	15 participantes (6 varones y 9 mujeres)	Edad promedio 17,2 ± 4,2a (13,9-26,2a)	Deficiencia transversal maxilar, hispánicos. Estadio de maduración vertebral ≥ CS4, biotipo dolicofacial	MSE (BioMaterials Korea, Inc)	2v/día hasta la apertura del diastema, después 1 v/día hasta conseguir el objetivo.	TCHC T0 y T2	La disyunción de la SMP fue paralela, con una separación de ENP del 90% respecto de la ENA (dirección sagital). Además, se registró una disyunción asimétrica (dirección transversal) a nivel de ENA, en promedio 1,1 mm (±1,0 mm), mayor movimiento en un lado que en el contralateral. Hubo una disyunción de la sutura pterigopalatina en el 53% pacientes entre las láminas mediales y laterales del proceso pterigoideo.
2	Cantarella *et al*. (^15^), EE. UU., 2018	Retrospectivo	15 participantes (6 varones y 9 mujeres)	Edad promedio 17,2 ± 4,2a (13,9-26,2a)	Deficiencia transversal maxilar Estadio de maduración vertebral ≥ CS4, biotipo dolicofacial	MSE (BioMaterials Korea, Inc) 4 DAT 1,5 mm diámetro con anclaje bicortical	2v/día hasta la apertura del diastema, después 1 v/día hasta conseguir el objetivo.	TCHC T0 y T2	Evaluación en el plano horizontal Medidas lineales: Se generó mayor movimiento lateral a nivel anterior intermaxilar, seguido de la distancia intercigomática (p < 0,01). A nivel intertemporal no hubo un movimiento estadísticamente significativo (p > 0,05). Medidas angulares: el ángulo de la apófisis cigomática del hueso temporal (más posterior) incrementó de forma significativa (p < 0,01), mientras que el ángulo cigomaticotemporal (más anterior) no tuvo incremento significativo estadísticamente (p > 0,05). Se deduce que el centro de rotación del complejo cigomaticomaxilar se ubica cerca de la porción proximal de la apófisis cigomático del hueso temporal. Se produce una flexión ósea a nivel de la apófisis cigomática del temporal durante el uso de MSE.
3	Cantarella *et al*. (^29^), EE. UU., 2018	Retrospectivo	15 participantes (6 varones y 9 mujeres)	Edad promedio 17,2 ± 4,2a (13,9-26,2a)	Deficiencia transversal maxilar, hispánicos. Estadio de maduración vertebral ≥ CS4, biotipo dolicofacial	MSE (BioMaterials Korea, Inc) 4 DAT 1,5 mm diámetro con anclaje bicortical	2v/día hasta la apertura del diastema, después 1 v/día hasta conseguir el objetivo.	TCHC T0 y T2	Evaluación en el plano coronal: Medidas lineales: distancia intercigomática superior e inferior incrementadas de forma significativa Medidas angulares: ángulo frontocigomático e inclinación maxilar aumentaron de forma significativa (p < 0,05). Ratio de 0.6° de desplazamiento del complejo craneofacial por cada mm de expansión a nivel intercigomatico inferior. Sin diferencia significativa a nivel de inclinación molar (p > 0,05). El completo cigomáticomaxilar rota hacia afuera con un centro de rotación localizado cerca a la sutura frontocigomática.
4	Cho *et al*. (^18^), Corea del Sur, 2022	Retrospectivo	23 participantes (10 varones y 13 mujeres)	Edad promedio 20,9 ± 3,65a (16-27a)	Deficiencia transversal maxilar -2	MSE (BioMaterials Korea, Inc) 4 DAT 1,8 x 11 mm con anclaje bicortical	<15a inicio 1 v/día ≥ 15a inicio 2 v/día > 20a inicio 4 v/día Después de apertura del diastema: 2 v/día en todas las edades hasta lograr la corrección.	TCHC T0 y T1	Todas las suturas evaluadas presentaron incremento en ancho: Sutura medio palatina (entre incisivos maxilares 3.06 ± 1,23 mm y ENP 2,52 ± 1,33 mm) S. intermaxilar (ENA 3,04 ± 1,13 mm) S. frontomaxilar (0,56 ± 0,38 mm) S. frontonasal (0,40 ± 0,16 mm) S. nasomaxilar (0,38 ± 0,21 mm)
5	Choi *et al*. (^6^), Corea, 2023	Retrospectivo	32 pacientes (14 varones y 18 mujeres)	Edad promedio 19,37a (12-29a)	Deficiencia transversal maxilar	MARPE: U46: Bandas en U4 y U6 (MSE-12 Biomaterials, Korea) y U6: Bandas en U6 (MSE-12, Biomaterials) ambos con apoyo en 4 DAT (1,5 x 11 mm y 1,5 x 13 mm) U46: cuerpo del tornillo a nivel de U5; U6 cuerpo del tornillo a entre U6 y U7.	2 v/día hasta conseguir la expansión deseada. Yonsei transverse index: -0,39 ± 1,87 mm)	Imágenes TCHC T0 y T2	Se reportaron diferencias significativas a nivel alveolar posterior y a nivel del hueso basal, siendo mayor en el tipo de expansor U6. Ambos presentaron una expansión piramidal en sentido coronal, con vértice hacia la zona frontonasal. Hubo una diferencia significativa en el número de DAT con anclaje bicortical, y fue mayor en el grupo U6.
6	Colak *et al*. (^16^), EE. UU., 2020	Retrospectivo	50 participantes (20 varones y 30 mujeres)	Edad promedio 18 ± 3a (10-27a)	Deficiencia transversal maxilar	MSE (BioMaterials Korea) con extensión y bandas en U6. 4 DAT con anclaje bicortical (mín. 11 mm)	02 a 4v/día (0,5-0,8 mm/día) hasta el diastema, después 1 v/día (0,25 mm) al día hasta completar activación	TCHC T0 y T2	Ángulo de apertura de la SMP fue de 0,57°, desde -80° (mayor apertura en ENP, hasta 1,30°). Expansión paralela 84% tuvo una disyunción de las láminas pterigoideas (medial y lateral) de las suturas pterigopalatinas derecha e izquierda (p < 0,01).
7	De Oliveira *et al*. (^26^), Brasil, 2021	Retrospectivo longitudinal	MARPE 17 pacientes (4 varones, 13 mujeres) SARPE 14 pacientes (6 varones, 10 mujeres)	MARPE: (15-37) edad promedio 22,9a, y SARPE: (18,7- 39,7) edad promedio 30,4a	Deficiencia transversal maxilar > 4 mm	MARPE con bandas en U6. 4 DAT 9 mm. SARPE con bandas en U4 y U6	MARPE: Activación 2/4 v/día (de 14-18 días) hasta lograr corrección. SARPE: Le Fort 1 osteotomía en pared lateral maxilar, sutura pterigopalatinar y sutura medio palatina y Hyrax: Activación 1/4 v/2 veces al día hasta lograr corrección	Imágenes TCHC	Se reportaron diferencias significativas en todas las mediciones esqueléticas con el MARPE (p < 0,05), incluso en el ancho facial medio y posterior, en donde la expansión con SARPE no registró una D. S. La expansión con SARPE tuvo una tendencia de expansión triangular tanto en el corte coronal como en el axial, teniendo como vértice la cavidad nasal y la espina nasal anterior, respectivamente; a diferencia de la expansión con MARPE, que obtuvo una tendencia más paralela con el MARPE. A nivel dental, se encontró que el aumento de las medidas se debió a una mayor inclinación dentaria y alveolar en sector posterior U6.
8	Elkenawy *et al.* (^31^), EE. UU., 2020	Retrospectivo	31 participantes	Edad promedio 20,4 ± 3,2a (17-27a)	Deficiencia transversal maxilar	MSE (BioMaterials Korea, Inc) extensión a bandas U6 y 4 DAT 1 x 5 x 11 mm	2 v/día hasta diastema 1 v/día hasta lograr objetivo	Modelos y TCHC	Expansión promedio de 4,98 mm (ANS). 4,77 (PNS) y 3,99 (Distancia sutura cigomático maxilar) (p < 0,001), 95,7% paralelismo (corte sagital). El 50% de la muestra tuvo una expansión asimétrica (promedio 2,22 mm más de un lado)
9	Lee *et al*. (^11^), Corea, 2021	Retrospectivo	18 participantes (6 v y 12 m) NG: 08 part sin apertura de sutura pterigopalatina SG: 10 part con apertura de sutura	Edad promedio 19,8 ± 4,8a (9-27a)	Deficiencia transversal maxilar	MSE II (BioMaterials, Korea), 4 DAT 1,5 x 11 mm	<15a inicio 1v/día ≥ 15a inicio 2v/día > 20a inicio 4v/día Después de apertura del diastema: 2 v/día en todas las edades hasta lograr la corrección	TCHC	Medias de expansión en ANS, PNS y centro de resistencia U6 entre NG y SG, diferencia significativa en la cantidad de expansión en PNS. NG: PNS expandió 71% con respecto a ANS SG: PNS expandió 111% con respecto a ANS. Además, NG 17/32 (30,6%) DAT no tuvieron anclaje bicortical, y en SG 5/40 (12,5%) de forma significativamente mayor falla en anteriores (p = 0,80). Correlación significativa entre anclaje bicortical y apertura de sutura pterigopalatina (p = 0,047).
10	Li *et al*. (^21^), China, 2020	Retrospectivo	48 pacientes (20 varones y 28 mujeres)	19,4 ± 3,3a	Deficiencia transversal maxilar > 3 mm	MSE Type II (BioMaterials Korea) con brazos soldados a bandas en U6. 4 DAT 1,5 x 11 mm G1: 4 DAT anclaje bicortical (n = 17) G2: 2 DAT anclaje bicortical posteriores (n = 17) G3: 4 DAT con anclaje en hueso cortical del paladar (n = 14)	1/6 vuelta al día hasta lograr objetivo. Retención: 3 meses	Imágenes TCHC	Todos los grupos registraron aumentos a nivel del ancho maxilar, nasal, del hueso zigomático, proceso pterigoideo (pterygoid plate) y hueso temporal. Este último, sin diferencia significativa en el grupo G3. El patrón de expansión fue piramidal en los tres grupos, corte coronal con la base a la altura del expansor. A nivel dentario, se registran cambios significativos en la pérdida de altura alveolar vestibular a nivel de molares en los 3 grupos (p < 0,05). También demostró una mayor inclinación vestibular del alveolo y los ejes de los dientes (p < 0,05).
11	McMullen *et al*. (^8^), EE. UU., 2022	Retrospectivo longitudinal	25 pacientes (13 hombres, 12 mujeres)	11,9-19,9a	Deficiencia transversal maxilar, 11 G1 (CVM 1-4/MSM B-C) y 14 G2 (CVM 5-6 y MSM D-E)	MSE con bandas U6. 4 DAT: 1,8 x 8 mm y 1,8 x 12 mm (anclaje bicortical)	Inició 2 semanas tras la instalación. Activación 8-13a inicio 2 v/día, 13-15a inicio 2-3 v/día, 16-17a inicio 3 v/día, 18a inicio 3-4 v/día. Después de apertura de diastema, 8-17a 2 v/día y 18a 2-3 v/día hasta conseguir corrección	Imágenes TCHC	Hubo un incremento de la dimensión transversal maxilar en GR en comparación con NG en NC y PF (p < 0,05). El ratio de expansión ósea-dentaria fue 62% ósea y 38% dentaria en GR y 59% ósea y 41% dentaria en NG. Leve desplazamiento hacia abajo y adelante (Or, Zyg, NC, U6, PNS, A-point ambos grupos). Cambios en la angulación hacia bucal de U6 en ambos grupos (4° GR y 3°NG) y de Caninos (3° GR y 1.5° NG).
12	Paredes *et al*. (^19^), EE. UU., 2020	Retrospectivo	39 participantes	Edad promedio 18,2 ± 4,2a (13,3-27,3a)	Deficiencia transversal maxilar	MSE extensión a bandas U6, 4 DAT 1,8 x 11 mm o 13 mm (bicortical)	2 v/día hasta la aparición del diastema, y 1 v/día hasta completar la activación	Modelos y TCHC	Medidas angulares: expansión esquelética 96,58% (derecha) y 95,44% (izquierda), 0,34 (derecha) y 0,33% (izquierda) flexión del hueso alveolar y 3,08% (derecha) y 4,23% (izquierda) inclinación dental. Medidas lineales: expansión esquelética 60,16% (derecha) y 56,83% (izquierda), 16,15 (derecha) y 16,55% (izquierda) flexión del hueso alveolar, 23,69% (derecha) y 26,62% (izquierda) inclinación dental.
13	Zong *et al.* (^27^), China y EE. UU., 2019	Retrospectivo	22 participantes (11 varones y 11 mujeres)	Edad promedio 14,97 ± 6,16a	Deficiencia transversal maxilar >4 y <10 mm	MSE (BioMaterials Korea) 04 DAT, 1,8 x 11 mm con anclaje bicortical	<13a inicio 2 v/día 13-15 inicio 2-3 v/día 16-17 inicio 3 v/día ≥ 18a inicio 3-4 v/día, Después de apertura del diastema: 2 v/día en todas las edades excepto ≥ 18a 2-3 v/día Hasta completar sobre expansión de 2-3 mm	TCHC T0 y T1	Expansión total U6 aumentó de forma significativa (5,41 ± 2,18 mm) (p < 0,001). Asimismo, el ancho maxilar (3,22 ± 1,63 mm) (p < 0,001). Además, un incremento en la inclinación bucal dentaria (2,56 ± 2,65°) (p < 0,001). Corte coronal: diferencia significativa siendo mayor en piso palatino con respecto al nasal (0,52 ± 0,11mm) (p < 0,001). Plano horizontal: sin diferencia significativa entre PNS y ANS. Expansión esquelética promedio (3,15 ± 1,64 mm) representó el 59,23% de la expansión total, y el resto fue dentaria (2,27 ± 1,25 mm), que representó el 40,96% de la expansión total.

a: años; v/día: vez al día; g: grupo; TCHC: tomografía computarizada de haz cónico; T0: antes de iniciar el tratamiento; T1: inmediatamente después de culminar las activaciones; T2: 3 semanas después de culminar las activaciones; T3: 3 meses después de culminar las activaciones; SMP: sutura media palatina; ENA: espina nasal anterior; ENP: espina nasal posterior ; DAT: dispositivo de anclaje temporal; U4: primera premolar superior; U6: primera molar inferior ; CVM: método de maduración cérvico-vertebral; MSM: maduración de sutura media palatina

## RESULTADOS

### Identificación de los estudios

Se identificaron 195 artículos en cuatro bases de datos: PubMed (n = 108), Scopus (n = 33), Embase (n = 24) y ScienceDirect (n = 30), hasta el 15 de junio de 2024. Se eliminaron 46 artículos duplicados con RefWorks y se descartaron 107 artículos por no cumplir los criterios de inclusión (n = 9); por ser reportes de caso (n = 11), revisiones (n = 8) o estudios en animales (n = 1); por no responder las preguntas de interés (n = 39) o por incluir expansores de diseños distintos (n = 39). Se obtuvieron 42 artículos, de los cuales fueron excluidos 29 por no cumplir con los criterios de selección (n = 28) y no incluir el texto completo (n = 1). Finalmente, se analizaron 13 artículos (Figura 1).

### Evaluación del riesgo de sesgo

**Figura 2:**
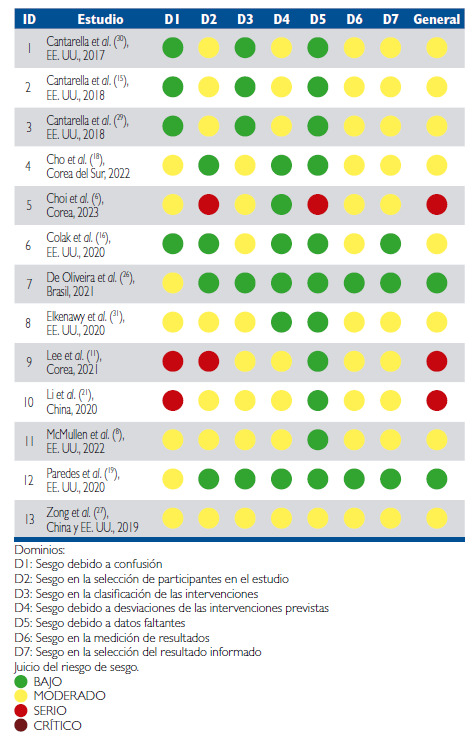
Evaluación de la calidad de los estudios (ROBINS-I)

Se utilizó la herramienta de ROBINS- I ^25^ para evaluar el riesgo de sesgo de los artículos seleccionados, los cuales fueron ensayos no aleatorizados. Se evaluaron siete dominios de sesgo: (D1) Sesgo debido a confusión, (D2) Sesgo en la selección de participantes en el estudio, (D3) Sesgo en la clasificación de las intervenciones, (D4) Sesgo debido a desviaciones de las intervenciones previstas, (D5) Sesgo debido a datos faltantes, (D6) Sesgo en la medición de resultados y (D7) Sesgo en la selección del resultado informado. Se realizó una evaluación general del sesgo (bajo, moderado, serio y crítico) para cada estudio incluido. Dos estudios ^(19, 26)^ presentaron riesgo de sesgo bajo. Ocho de ellos ^(8, 15, 16, 18, 27, 29, 30, 31)^, riesgo moderado. Tres de los estudios ^(6, 11, 21)^ mostraron riesgo de sesgo severo debido a factores de confusión, selección de participantes y datos faltantes. No se hallaron estudios con riesgo de sesgo crítico; por ende, no hubo necesidad de eliminar ninguno (Figura 2)

Dominios: D1: Sesgo debido a confusión D2: Sesgo en la selección de participantes en el estudio D3: Sesgo en la clasificación de las intervenciones D4: Sesgo debido a desviaciones de las intervenciones previstas D5: Sesgo debido a datos faltantes D6: Sesgo en la medición de resultados D7: Sesgo en la selección del resultado informado Juicio del riesgo de sesgo. BAJO MODERADO SERIO CRÍTICO

### Características demográficas y características clínicas de la muestra

Todos los estudios fueron realizados en 4 poblaciones: Brasil ^26^, China ^21^^,^^27^ Corea ^6^^,^^18^^,^^28^ y EE. UU. ^8^^,^^15^^,^^16^^,^^19^^,^^27^^,^^29^^-^^31^. En esta última ubicación, algunos estudios indicaron evaluar específicamente población hispana ^15^^,^^29^^,^^30^.

Todos los estudios tuvieron un diseño retrospectivo. El rango de muestra osciló entre 15 y 50 participantes por estudio, cuyas edades fueron desde los 9 hasta los 37 años. Todos los estudios tuvieron como principal criterio de inclusión la deficiencia transversal maxilar mínima de 2-3 mm, la cual fue evaluada en los siguientes modelos: análisis de los seis elementos de Andrews ^26^, una modificación del mismo análisis ^15^^,^^16^^,^^29^^,^^30^^,^^31^ o en CBCT, utilizando la distancia entre los puntos más cóncavos del vestíbulo maxilar para el ancho del hueso maxilar y la distancia entre la corteza vestibular a nivel del surco mesiovestibular de L6 para el ancho mandibular ^21^. También se utilizó el método de Betts ^27^ y la diferencia de las distancias entre los centros de resistencia de primeras molares maxilares y mandibulares menores a 2 mm ^18^. Todos los estudios evaluaron los efectos y cambios estructurales en CBCT, y utilizaron un diseño similar de MARPE, el cual consta de un tornillo central con 4 tubos soldados que sirven de guía para la inserción de los microimplantes en la zona para medial al rafe medio del paladar, los cuales deben alcanzar un anclaje tanto de la cortical palatina como de la nasal. Además, el tornillo central consta de brazos soldados a bandas en U6, lo cual brinda una mayor estabilidad. 

### Cambios y efectos esqueléticos

Todos los estudios evaluaron cambios esqueléticos generados en el complejo maxilofacial con el uso de MARPE. La cantidad de participantes estudiados osciló entre 15 y 50 pacientes. Todos los estudios utilizaron CBCT para planificar el tratamiento y evaluar los cambios estructurales al finalizar la expansión, antes de colocar aparatología fija. 

Se reportan 9 suturas involucradas en el complejo craneofacial durante la expansión maxilar asistida con microimplantes en pacientes con bajo o nulo crecimiento residual: frontonasal, frontomaxilar, frontocigomática, nasomaxilar, cigomáticomaxilar, intermaxilar, palatina media, cigomáticotemporal y pterigopalatina ^6^^,^^18^.

Solo uno de los estudios evalúa los cambios en ancho de las 9 suturas y encontró aumentos en el ancho estadísticamente significativos en todas ellas tras la expansión con MARPE ^18^.

Ocho estudios evaluaron el esquema de expansión anteroposterior en el corte axial de la SMP ^6^^,^^16^^,^^18^^,^^26^^-^^28^^,^^30^^,^^31^. El patrón fue paralelo o casi paralelo en la mayoría de los estudios; algunos utilizaron medidas lineales entre el sector anterior (3,07 +/- 1,57 mm) y el posterior (3,28 +/- 1,68 mm) del paladar tras la expansión ^27^. Otros evaluaron el patrón de disyunción con medidas angulares y encontraron también una tendencia paralela en donde el promedio de apertura de la sutura media palatina fue de 0,57° (-0,80° hasta 1,30°) con vértice posterior ^16^.

Además de reportar una disyunción a nivel de espina nasal posterior (ENP) de 2,52 mm, que representa el 82% con respecto a la espina nasal anterior (ENS) de 3,04 mm, Cho *et al*. ^18^ hallaron que la separación entre los incisivos centrales maxilares (3,06 mm) constituye el 101% de expansión con respecto a ENA. 

Uno de los estudios comparó el paralelismo axial entre MARPE y SARPE, y encontró en ambos una expansión mayor en anterior que posterior (p < 0,05), aunque con tendencia más paralela en MARPE (-0,95 mm) en comparación con SARPE (-1,65 mm).

Dos estudios ^30^^,^^31^ describen que la separación a nivel de la ENP representó, en promedio, el 90% ^29^ o incluso el 95% ^31^ con respecto a ENA. Ambos estudios ^30^^,^^31^ evalúan incluso la simetría en la disyunción del sector anterior (ENA) entre el lado derecho e izquierdo tras la expansión. Elkenawy *et al*. ^31^ encuentran que el 51% de su muestra presenta una asimetría estadísticamente significativa de 1,1 mm, en promedio, entre el lado derecho e izquierdo tras la expansión con MSE. Cantarella *et al*. ^30^ describen una asimetría similar en la expansión a nivel de ENA de 1,1 mm (+/-1 mm) entre un lado y otro.

Lee *et al*. ^28^ describen un esquema de expansión piramidal de la SMP donde, a nivel de ENP, la separación representó el 71% de la expansión con respecto a ENA (p = 0,036) en el grupo donde no hubo apertura de la sutura pterigopalatina. También hallaron una expansión en ENP del 111% con respecto a ENA en el grupo que presentó disyunción a nivel de la misma sutura ^28^.

Cantarella *et al*. ^15^ refieren que el centro de rotación (CR) axial se encuentra cercano a la parte proximal de la apófisis cigomática del hueso temporal. Además, describe un desplazamiento significativo del tercio medio (ancho maxilar anterior, hueso y arco cigomáticos) tras la expansión con MSE ^27^. En ese sentido, otros estudios también describen en específico incrementos a nivel del ancho maxilar tras la expansión: 3.22 +/- 1,63 mm (p < 0,001) ^27^, y en el ancho de la sutura nasofrontal, cigomático maxilar y ancho de la cavidad nasal ^6^. Oliveira *et al*. ^26^ incluso resaltan que los cambios esqueléticos en el tercio medio facial son significativamente mayores en MARPE en comparación con SARPE (p < 0,05). 

Dos estudios ^8^^,^^28^ encuentran desplazamiento hacia adelante de la ENA. Uno de ellos, al comparar los efectos de MARPE entre pacientes en crecimiento y sin crecimiento, refiere un desplazamiento mayor hacia adelante y debajo de la ENA en pacientes con crecimiento residual (1,1 +/- 0,6 mm), en comparación con los que no (NG 0,5 +/- 0,5 mm) (p = 0,004) ^8^. El otro describe un mayor desplazamiento hacia anterior en pacientes sin crecimiento residual cuya sutura pterigopalatina fue desarticulada tras la expansión (0,06 +/- 0,64 mm), a diferencia del grupo que no tuvo cambios a nivel de dicha sutura (0,89 +/- 0,49 mm) ^28^.

Tres estudios evalúan la desarticulación de la sutura pterigopalatina. Uno halla que en el 53% de la muestra existe una disyunción específicamente entre las láminas mediales y laterales del proceso pterigoideo (p < 0,01) ^30^. Otro refiere una separación completa en el 84% y parcial en el 16% de las suturas en pacientes sin crecimiento residual ^16^. Por otro lado, Lee *et al*. ^28^ describen que el 55,6% de la muestra presenta apertura de la sutura pterigopalatina y encuentra una correlación significativa entre el anclaje bicortical y la apertura de esta sutura (p = 0,0003).

En el corte coronal, dos estudios evalúan el centro de rotación del complejo cigomático maxilar. El primero concluye que se encuentra en el punto más distal de la distancia interfrontal (línea que une los puntos más externos e inferiores de los procesos cigomáticos del hueso frontal) o levemente superior a la distancia antes descrita ^19^. El segundo señala que el centro de rotación se ubica levemente por encima de la sutura frontocigomática. Conjuntamente, encuentran que, por cada milímetro de expansión a nivel de la distancia intercigomática inferior (punto más bajo de la sutura cigomático maxilar), cada mitad del complejo cigomático maxilar rota 0,6° sobre el fulcrum descrito ^29^. Esto justificaría la tendencia de expansión piramidal con vértice superior en el corte coronal de algunos estudios ^6^^,^^19^^,^^21^^,^^26^^,^^27^^,^^29^.

### Cambios y efectos dentoalveolares

Siete artículos estudian los efectos a nivel dentoalveolar. Dado que el diseño del MSE tiene extensiones de alambre que se apoyan en U6, estas son las piezas principalmente evaluadas. La inclinación molar tras la expansión no tuvo diferencia significativa en cuatro estudios, y presentó una inclinación hacia bucal de 3,3° en promedio bilateral ^26^, 2,04° al lado derecho y 1,83° al lado izquierdo ^29^, 0,09° al lado derecho y 0,013° al lado izquierdo ^19^, con 2,56° +/- 2,65° en promedio ^27^.

Choi *et al*. ^6^ describen cambios en la inclinación molar de 7,06° tras la expansión en el diseño de MSE con apoyo en primeros molares maxilares, en comparación con un diseño de MARPE cuyo apoyo no solo se encontraba en U6, sino también en primeros premolares maxilares (U4), donde la inclinación fue de 5,88° tras la expansión.

Por otro lado, Li *et al*. ^21^ evalúan los cambios dentarios tras la expansión en relación con la bicorticalidad del anclaje, y hallaron que existe una menor expansión a nivel de ápices palatinos de U6 y una mayor inclinación bucal de los alveolos y ejes dentarios en el grupo de 4 microimplantes sin anclaje bicortical, en comparación con los grupos con 2/4 o 4/4 microimplantes con anclaje bicortical (p < 0,05).

McMullen *et al*. ^8^ compararon la inclinación de primeros molares maxilares tras la expansión entre pacientes en crecimiento (GR) y pacientes sin crecimiento residual (NG), y encontraron una inclinación bucal promedio de 4° y 3° en pacientes GR y NG, respectivamente. Además, reportaron una diferencia significativa en cuanto al desplazamiento inferior de los caninos (U3) (p = 0,02), de 1,7 +/- 1 mm en el grupo en crecimiento y 0,6 +/- 0,8 mm en el grupo sin crecimiento residual.

## DISCUSIÓN

La finalidad de este estudio es dar a conocer los cambios estructurales generados en el complejo craneofacial tras la expansión maxilar asistida con microimplantes tipo MSE en pacientes con deficiencia transversal del maxilar, mediante una revisión exhaustiva de artículos que cumplían con los criterios de selección preestablecidos. 

Todos los estudios demuestran que es fundamental el uso de CBCT para la planificación (MARPE) y para evaluar los cambios en el complejo craneofacial tras la expansión. Sin embargo, no todos utilizan este método (CBCT) para el diagnóstico de deficiencia transversal maxilar; en su mayoría utilizan la clasificación en modelos de estudio ^15^^,^^16^^,^^26^^,^^29^^-^^31^.

Todos los estudios demuestran cambios significativos a nivel del complejo craneofacial tras la expansión con MARPE/MSE. Son 9 las suturas involucradas durante la expansión con MARPE ^18^, de las cuales la sutura media palatina, cigomáticomaxilar y pterigopalatina son las que presentan mayor resistencia a la expansión ^17^^,^^18^^,^^21^.

La mayor proporción de disyunción de la SMP se reporta en el estudio de Cantarella *et al*. del 67% en promedio ^30^, en comparación con Zong *et al*. ^27^, quienes encuentran un ratio del 59,23% en promedio de expansión esquelética y el 40,96% en dentaria. A su vez, Elkenawy *et al*. ^8^ encuentran resultados similares entre pacientes en crecimiento: un 62% esquelético y un 38% dentario, al ser comparados con pacientes sin crecimiento residual (59% esquelético y 41% dental). Estos hallazgos sustentarían el uso de MARPE incluso en pacientes con crecimiento residual en donde se requiera disminuir efectos adversos de RPE (inclinaciones bucales de los dientes), a fin de obtener un mayor efecto esquelético.

Respecto del esquema de disyunción de la SMP, la mayoría de los estudios encuentran un patrón de expansión paralela cuando se usa MARPE, en donde, a nivel de ENP, la expansión representa el 82%, 90% y 95,7% con relación a ENA ^18^^,^^30^^,^^31^. Colak *et al*. ^16^^)^ refieren que es la sutura pterigopalatina la estructura que dictamina el patrón de expansión axial, ya que limita la expansión posterior de la SMP. Lee *et al*. ^28^ detallan que, para lograr la disyunción de dicha sutura, es necesario obtener al menos anclaje bicortical en los microimplantes posteriores, y que es ideal el anclaje bicortical de los anteriores. Esto lo justifica al encontrar una correlación significativa entre el anclaje bicortical de los microimplantes y la apertura de la sutura pterigopalatina (p = 0,0003). De esta forma, se podría garantizar un patrón de expansión de la SMP más paralelo. 

Asimismo, Li *et al*. ^21^ señalan que, además de aumentar la posibilidad de la expansión axial paralela, la bicorticalidad de al menos los microimplantes posteriores generaría una expansión menos piramidal en el corte coronal, un mayor incremento de la distancia intercigomática e, incluso, del ancho del hueso temporal (lo cual afectaría de forma directa la posición de los cóndilos con respecto a la fosa articular). No se encuentran más investigaciones al respecto. 

Dos estudios ^30^^,^^31^ encuentran, a nivel de espina nasal anterior, una asimetría de 1,1 mm en promedio tras la expansión con MARPE, lo cual podría deberse a la densidad de las suturas o huesos, la estabilidad de los microimplantes o, incluso, al mismo patrón inicial de mordida cruzada (uni o bilateral). Ambos estudios ^30^^,^^31^ consideran la evaluación de asimetrías a nivel de ENA, pues podría representar cierta repercusión en el tejido blando.

Con relación al patrón de expansión en el corte coronal, esta investigación encuentra una tendencia de expansión piramidal con vértice superior, lo cual se justifica porque el fulcrum de rotación del diseño evaluado se localiza cerca de la sutura frontocigomática ^19^^,^^29^. Cantarella *et al*. ^29^ reportan que, por cada milímetro de expansión a nivel de la SMP, la rotación del complejo cigomático maxilar sobre el fulcrum sería de 0,6°. Por ende, se encuentran cambios considerables en todo el tercio medio facial tras la expansión maxilar asistida con microimplantes (ancho nasal, distancia intercigomática, hueso palatino), lo que aumenta conforme nos alejamos del fulcrum. 

De los seis artículos que evalúan movimientos dentarios tras la expansión maxilar asistida con microimplantes: 5 encuentran que la inclinación bucal de las molares no tiene repercusión clínica (desde 0,09° hasta 3,3°) ^19^^,^^21^^,^^27^^,^^26^^,^^29^, incluso en el estudio que evalúa pacientes con y sin crecimiento residual (4° y 3° en promedio, respectivamente) ^8^. Uno de ellos, sin embargo, encuentra que el diseño de MSE con extensiones y apoyo en U6 inclina de forma significativa las primeras molares maxilares (7,06°) hacia bucal tras la expansión con MARPE ^6^.

### Limitaciones

Todos los estudios incluidos fueron retrospectivos, lo cual generó cierta limitación al analizar o buscar correlación entre interrogantes que podían surgir. Las muestras evaluadas oscilaban entre 15 a 50 participantes por estudio, se podrían realizar estudios en poblaciones mayores. Asimismo, se evaluaron pacientes de diferente etnia y patrones esqueléticos, lo cual podría limitar el generalizar los efectos esqueléticos y dentoalveolares encontrados. 

## CONCLUSIONES

Es posible realizar correcciones transversales del tercio medio facial en pacientes con y sin crecimiento residual, a la espera de disminuir los efectos adversos. Se debería ampliar la evaluación a pacientes de diversas etnias y patrones esqueléticos para buscar conformidad entre los resultados. La investigación evalúa los cambios estructurales generados en el complejo craneofacial tras la expansión maxilar asistida con microimplantes con un diseño específico de expansor. Por ende, no sería posible generalizar los hallazgos estructurales a todas las variaciones de MARPE que existen en la actualidad.

## Financiamiento:

Autofinanciado

## References

[1] Brunelle JA, Bhat M, Lipton JA (1996). Prevalence and distribution of selected occlusal characteristics in the U S. population, 1988-1991. J Dent Res.

[2] Asiri SN, Tadlock LP, Buschang PH (2019). The prevalence of clinically meaningful malocclusion among US adults. Orthod Craniofacal Res.

[3] McNamara JA (2000). Maxillary transverse deficiency. Am J Orthod Dentofacial Orthop.

[4] Ugolini A, Agostino P, Silvestrini-Biavati A, Harrison JE, Batista KB (2021). Orthodontic treatment for posterior crossbites. Cochr Datab Syst Rev.

[5] Kapetanovic A, Theodorou CI, Bergé SJ, Schols JGJH, Xi T (2021). Efficacy of Miniscrew-Assisted Rapid Palatal Expansion (MARPE) in late adolescents and adults a systematic review and meta-analysis. Eur J Orthod.

[6] Choi HY, Lee SM, Lee JW, Chung DH, Lee MH (2023). Skeletal and dentoalveolar effects of different types of microimplant-assisted rapid palatal expansion. Korean J Orthod.

[7] Angell EC (1860). Treatment of irregularities of the permanent or adult teeth. Dental Cosmos.

[8] McMullen C, Al Turkestani NN, Ruellas ACO, Massaro C, Rego MVNN, Yatabe MS, Kim-Berman H, McNamara JA, Angelieri F, Franchi L, Ngan P, He H, Cevidanes LHS (2022). Three-dimensional evaluation of skeletal and dental effects of treatment with maxillary skeletal expansion. Am J Orthod Dentofacial Orthop.

[9] Chamberland S, Proffit WR (2011). Short-term and long-term stability of surgically assisted rapid palatal expansion revisited. Am J Orthod Dentofacial Orthop.

[10] (2012). Complications following surgically assisted rapid palatal expansion a retrospective cohort study. J Oral Maxillofac Surg.

[11] Lee KJ, Park YC, Park JY, Hwang WS (2010). Miniscrew-assisted nonsurgical palatal expansion before orthognathic surgery for a patient with severe mandibular prognathism. Am J Orthod Dentofacial Orthop.

[12] Jin B, Jin-Young C, Seong-Hun K (2024). The design of bone-borne maxillary expander affects the different dentoalveolar inclination and expansion pattern A CBCT study, Seminars in. Orthodontics.

[13] Tausche E, Hansen L, Hietschold V, Lagravere MO, Harzer W (2007). Three-dimensional evaluation of surgically assisted implant bone-borne rapid maxillary expansion a pilot study. Am J Orthod Dentofacial Orthop.

[14] MacGinnis M, Chu H, Youssef G, Wu KW, Machado AW, Moon W (2014). The effects of micro-implant assisted rapid palatal expansion (MARPE) on the nasomaxillary complex--a finite element method (FEM) analysis. Prog Orthod.

[15] Cantarella D, Dominguez-Mompell R, Moschik C, Sfogliano L, Elkenawy I, Pan HC, Mallya SM, Moon W (2018). Zygomaticomaxillary modifications in the horizontal plane induced by micro-implant-supported skeletal expander, analyzed with CBCT images. Prog Orthod.

[16] Colak O, Paredes NA, Elkenawy I, Torres M, Bui J, Jahangiri S, Moon W (2020). Tomographic assessment of palatal suture opening pattern and pterygopalatine suture disarticulation in the axial plane after midfacial skeletal expansion. Prog Orthod.

[17] Suzuki H, Moon W, Previdente LH, Suzuki SS, Garcez AS, Consolaro A (2016). Miniscrew-assisted rapid palatal expander (MARPE): the quest for pure orthopedic movement. Dental Press J Orthod.

[18] Cho AR, Park JH, Moon W, Chae JM, Kang KH (2022). Short-term effects of microimplant-assisted rapid palatal expansion on the circummaxillary sutures in skeletally mature patients A cone-beam computed tomography study. Am J Orthod Dentofacial Orthop.

[19] Paredes N, Colak O, Sfogliano L, Elkenawy I, Fijany L, Fraser A, Zhang B, Moon W (2020). Differential assessment of skeletal, alveolar, and dental components induced by microimplant-supported midfacial skeletal expander (MSE), utilizing novel angular measurements from the fulcrum. Prog Orthod.

[20] Siddhisaributr P, Khlongwanitchakul K, Anuwongnukroh N, Manopatanakul S, Viwattanatipa N (2022). Effectiveness of miniscrew assisted rapid palatal expansion using cone beam computed tomography A systematic review and meta-analysis. Korean J Orthod.

[21] Li N, Sun W, Li Q, Dong W, Martin D, Guo J (2020). Skeletal effects of monocortical and bicortical mini-implant anchorage on maxillary expansion using cone-beam computed tomography in young adults. Am J Orthod Dentofacial Orthop.

[22] Moon HW, Kim MJ, Ahn HW, Kim SJ, Kim SH, Chung KR, Nelson G (2020). Molar inclination and surrounding alveolar bone change relative to the design of bone-borne maxillary expanders A CBCT study. Angle Orthod.

[23] Choi JY, Choo H, Oh SH, Park JH, Chung KR, Kim SH (2021). Finite element analysis of C-expanders with different vertical vectors of anchor screws. Am J Orthod Dentofacial Orthop.

[24] Page MJ, McKenzie JE, Bossuyt PM, Boutron I, Hoffmann TC, Mulrow CD (2021). The PRISMA 2020 statement an updated guideline for reporting systematic reviews. BMJ.

[25] Sterne JAC, Hernán MA, Reeves BC, Savovic J, Berkman ND, Viswanathan M, Henry D, Altman DG, Ansari MT, Boutron I, Carpenter JR, Chan AW, Churchill R, Deeks JJ, Hróbjartsson A, Kirkham J, Jüni P, Loke YK, Pigott TD, Ramsay CR, Regidor D, Rothstein HR, Sandhu L, Santaguida PL, Schünemann HJ, Shea B, Shrier I, Tugwell P, Turner L, Valentine JC, Waddington H, Waters E, Wells GA, Whiting PF, Higgins JPT (2016). ROBINS-I: a tool for assessing risk of bias in non-randomized studies of interventions. BMJ.

[26] de Oliveira CB, Ayub P, Ledra IM, Murata WH, Suzuki SS, Ravelli DB, Santos-Pinto A (2021). Microimplant assisted rapid palatal expansion vs surgically assisted rapid palatal expansion for maxillary transverse discrepancy treatment. Am J Orthod Dentofacial Orthop.

[27] Zong C, Tang B, Hua F, He H, Ngan P (2019). Skeletal and Dentoalveolar Changes in the Transverse Dimension using Microimplant-Assisted Rapid Palatal Expansion (MARPE) Appliances. Semin Orthod. 2019.

[28] Lee DW, Park JH, Moon W, Seo HY, Chae JM (2021). Effects of bicortical anchorage on pterygopalatine suture opening with microimplant-assisted maxillary skeletal expansion. Am J Orthod Dentofacial Orthop.

[29] Cantarella D, Domínguez-Mompell R, Moschik C, Mallya SM, Pan HC, Alkahtani MR, Elkenawy I, Moon W (2018). Midfacial changes in the coronal plane induced by microimplant-supported skeletal expander, studied with cone-beam computed tomography images. Am J Orthod Dentofacial Orthop.

[30] Cantarella D, Domínguez-Mompell R, Mallya SM, Moschik C, Pan HC, Miller J, Moon W (2017). Changes in the midpalatal and pterygopalatine sutures induced by micro-implant-supported skeletal expander, analyzed with a novel 3D method based on CBCT imaging. Prog Orthod.

[31] Elkenawy I, Fijany L, Colak O, Paredes NA, Gargoum A, Abedini S, Cantarella D, Domínguez-Mompell R, Sfogliano L, Moon W (2020). An assessment of the magnitude, parallelism, and asymmetry of micro-implant-assisted rapid maxillary expansion in non-growing patients. Prog Orthod.

